# Tongue Contour Tracking and Segmentation in Lingual Ultrasound for Speech Recognition: A Review

**DOI:** 10.3390/diagnostics12112811

**Published:** 2022-11-15

**Authors:** Khalid Al-hammuri, Fayez Gebali, Ilamparithi Thirumarai Chelvan, Awos Kanan

**Affiliations:** 1 Department of Electrical and Computer Engineering, University of Victoria, Victoria, BC V8W 2Y2, Canada; 2 Department of Computer Engineering, Princess Sumaya University for Technology, Amman 11941, Jordan

**Keywords:** tongue contour tracking, medical imaging analysis, computer vision, lingual ultrasound, machine learning, image segmentation

## Abstract

Lingual ultrasound imaging is essential in linguistic research and speech recognition. It has been used widely in different applications as visual feedback to enhance language learning for non-native speakers, study speech-related disorders and remediation, articulation research and analysis, swallowing study, tongue 3D modelling, and silent speech interface. This article provides a comparative analysis and review based on quantitative and qualitative criteria of the two main streams of tongue contour segmentation from ultrasound images. The first stream utilizes traditional computer vision and image processing algorithms for tongue segmentation. The second stream uses machine and deep learning algorithms for tongue segmentation. The results show that tongue tracking using machine learning-based techniques is superior to traditional techniques, considering the performance and algorithm generalization ability. Meanwhile, traditional techniques are helpful for implementing interactive image segmentation to extract valuable features during training and postprocessing. We recommend using a hybrid approach to combine machine learning and traditional techniques to implement a real-time tongue segmentation tool.

## 1. Introduction

The main objective of this review is to evaluate existing methodological approaches for tongue contour tracking using ultrasound images in speech recognition applications. The paper also describes research insights, existing gaps, and future research directions [1]. We consider the mean sum of distances (MSD) as the primary evaluation criterion for the quantitative analysis of tongue segmentation. MSD is the standard measure of tongue segmentation in research as it considers the variation of tongue length, and it is adopted widely in tongue segmentation publications. For the qualitative analysis, we consider algorithm usability, image quality, and the shape consistency of the segmented tongue contour.

Studying tongue movement during speech is essential to the understanding of human articulation. Different approaches are used to study speech; some rely on a single sensor [2,3,4,5,6], and others use hybrid techniques [7,8,9]. Due to medical imaging modalities advancement and impressive capabilities, linguistic researchers are relying on the medical ultrasound system to capture tongue motion during speech [10]. Ultrasound imaging is considered the most efficient methodology in terms of safety and portability. However, magnetic resonance imaging (MRI) has a better resolution, and it can provide more information about the soft tissues [11], vocal tract, and craniofacial structure [12,13]. MRI is used for real-time image acquisition [11,14,15] to visualize the vocal tract either in 2D or 3D orientation [16,17] and enhance the speech analysis. However, MRI is huge in size and very expensive compared to ultrasound. It requires a special arrangement and a long scanning time, making it impractical for most of the day-to-day uses of speech analysis to limit its application for particular research or clinical studies.

On the other hand, X-ray [18,19,20] and CT [21,22,23,24,25] systems are cheaper than MRI, they have a reasonable resolution, and they have many applications as well. X-ray is used for tongue contour extraction [26,27]; it is also used for tongue contour image synthesis to create articulation copy [28] or combining physiological models to fit X-ray images. An X-ray system is also beneficial for capturing images of the whole vocal tract [29] and nonrigid articulatory structures [30]. CT scan has a wide variety of applications compared to conventional X-rays. CT scan is used in clinical studies of oral-cavity-related disorders such as sleep apnea [22,31]. CT images are also used to estimate the tongue volume within the oral cavity [32,33,34,35]. Furthermore, CT is applied in advanced surgical procedures as it is beneficial for image registration [36]; augmented reality and CT images are also combined to guide transoral robotic surgery [37]. In addition, CT-mapped 3D images of different tongue types have been used in clinical applications of tongue cancers [38]. However, CT and X-rays are larger in size compared to ultrasound, and they have a radiation danger which requires a strictly yearly radiation dose limit to prevent harmful radiation for humans. At the same time, ultrasound is safer and has no radiation danger to the user.

In addition to the medical imaging systems, biosignal sensors are also utilized for speech analysis and related studies. Types of biosignal sensors [8] are electromagnetic articulatory (EMA), permanent magnet articulography (PMA), electropalatography (EPG), electromyography (EMG), electroencephalography (EEG).

EMA [39,40,41] is useful to localize the movement within the vocal tract by using electromagnetic transmitter coils to track the position of the attached electromagnetic sensors on the tongue, lips, and jaw. EMA may provide either a 2D or 3D landmark localization in milliseconds, but the system operation is complex and uncomfortable to be used in all cases on a daily basis; it might be more usable for conducting clinical studies at research centres. In ultrasound research, EMA data are used to build a prediction machine learning model to guide ultrasound tracking to minimize the effect of missing data.

On the other hand, PMA [42,43,44,45,46] is a technique to capture articulator displacement by using a permanent magnet on the tongue and detecting the magnetic signal using a wearable sensor. It is useful for speech recognition tasks, and the reported word detection accuracy is around 90% [42]. Unlike EMA, PMA does not have wires and has a reverse transmitter–receiver arrangement to make it more convenient [8]. However, PMA sensors configuration is not convenient, and it is difficult to maintain the same position reference for all cases.

EPG is used for tongue tracking and speech therapy [8,47]. Moreover, EPG information is also applied to get an accurate image registration by a CT scanner [35]. Furthermore, EPG can be combined with audio signals for speech generation and speech enhancement applications [48]. EPG uses a hard plate beneath the tongue to detect the contact between the tongue and the array of sensors in the plate. The hard plate requires a specialized dentist to get a measure as it should be custom-designed for each patient. However, EPG can give some information about the tongue motion, but it is not practical, and limited data can be acquired from it compared to ultrasound.

EMG for speech recognition [49,50,51] is more convenient and safer than EMA, PMA and EPG as it uses surface electrodes on the face without any invasive measures. EMG is a system that detects the muscles’ electric activity and its nerves’ biosignals [52]. The detected signals can give an indication of the muscles’ health [53]. However, in the case of speech recognition, the muscles’ movement can indicate the speech behaviour and its relationship with the tongue muscle motion [54]. Moreover, EMG can be used to translate hand gestures for a speech to help people with speech impediments [55].

Studying brain electrical activity using EEG is useful for speech analysis. The acoustic sound stimulates the auditory cortex in the brain which generates electrical signals that can be detected by the electrodes or small metal plates attached to the scalp. Different research studies have proposed to analyze EEG signals and extract the relationship between brain signals and speech behaviour [56,57,58,59]. Although EEG can provide information about the speech patterns, the nature of the EEG signal is complex and susceptible to noise, which makes the part of the EEG complex signal relating to the auditory system difficult to be separated from other electrical activities of the brain [8,60]. Many advanced techniques have been proposed to alleviate this issue by proposing artifact removal [61,62] or incorporating advanced deep learning techniques such as a Transformer model and a generative adversarial network analysis [63,64].

The remaining of the article is organized as follows. Section 2 provides an overview of ultrasound imaging in speech recognition. Section 3 describes the standard evaluation measures of the tongue segmentation algorithms. Section 4 includes the tongue contour tracking techniques in ultrasound images. Section 5 discuss the algorithms quantitative and qualitative evaluation results. Finally, conclusion provided in Section 6.

## 2. Overview of Ultrasound Imaging in Speech Recognition

An ultrasound system is portable, safe, and convenient, making it efficient for real-time image acquisition inside or outside hospitals. Researchers and clinical linguists have widely adopted the use of lingual ultrasound for different applications. Some of these applications include using it as a visual feedback for second language teaching [65,66], speech remediation to correct articulation for people with speech disabilities [67], speech-related disorders such as autism [4,68,69], articulation research and analysis [10,65,70], swallowing studies [71], tongue 3D modelling [72], and silent speech interface [7,73,74,75]. Furthermore, ultrasound imaging analysis is used in many applications in medical imaging analysis for object detection and segmentation. Some of these applications are in the field of cardiology, in which researchers obtain echocardiography images for the heart to help cardiologists identify the health status of the heart [76,77]. Echocardiography image segmentation is beneficial for measuring the left ventricle volume and estimating its blood ejection fraction. It is also useful for examining heart valve performance. Moreover, ultrasound is also one of the safest and most efficient tools for studying breast cancer and assisting with cancer biopsy. Ultrasound images could help physicians examine breast tissues to identify if a cancerous mass is benign or malignant, either in two-dimension (2D) images [76,78,79] or three-dimension (3D) images [80]. A portable ultrasound system is also used in healthcare facilities to assist in intravascular procedures [81,82]. Obstetrics and gynecology use ultrasound systems on a daily basis to examine and mentor pregnant women’s health and fetus growth [83,84]. Furthermore, ultrasound is also used to detect ovarian tumours, which is one of the main diseases that affect women’s health [85].

**Figure 1:**
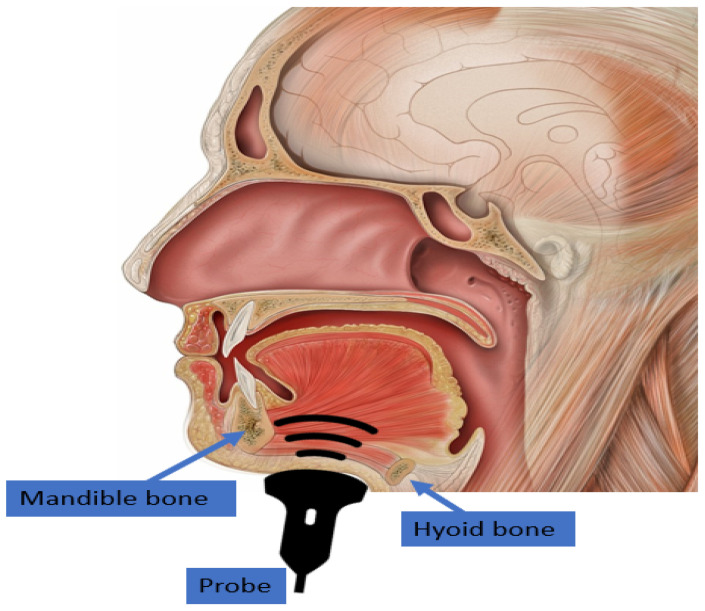
Overview of ultrasound probe placement beneath the chin. The ultrasound wave is shown in a black arc generated from the acoustic probe and propagated in the direction of the tongue. The effect of the hyoid and mandible bones is blocking part of the ultrasound wave, as shown in a black colour. The head and oral cavity picture was modified from the original picture for the case, courtesy of Associate Professor Frank Gaillard, Radiopaedia.org, rID: 35836, [86].

Figure 1 visualizes the placement of the ultrasonic transducer beneath the chin and the propagation of the acoustic wave. To capture the tongue image, an ultrasound transducer should be placed beneath the chin during the image acquisition to acquire the most applicable view of the tongue contour. Ultrasound waves pass through the chin tissues in-between the hyoid and mandible bones to reach the tongue. The impedance mismatch between the tongue tissue and the air causes a strong reflection of the acoustic waves, which allows us to detect the tongue structure by detecting the reflected acoustic waves. However, the tongue is positioned deeply in the oral cavity, making it challenging to fully view the contour during sound production. The hyoid and mandible bones absorb some acoustic waves, which may block the view of the tongue tip and root. Moreover, the shadowing of jawbones and instability of the head-transducer position would add other obstacles to the experiment.

**Figure 2:**
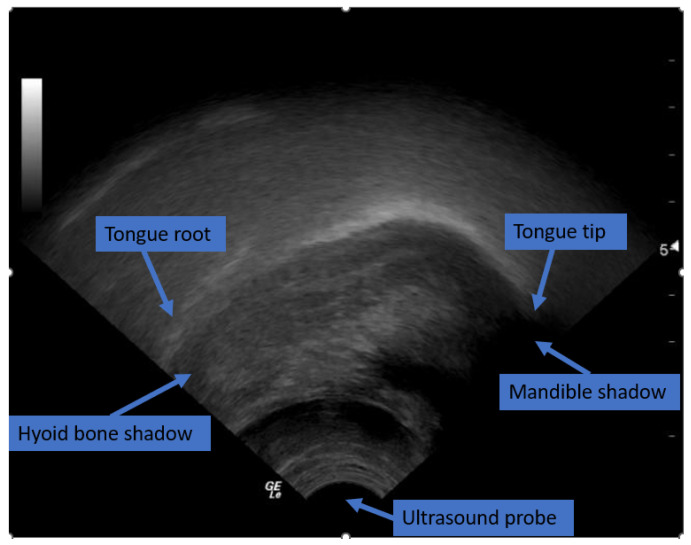
Ultrasound image of the tongue showing the tongue tip and root in the sagittal plane. The ultrasound probe on the bottom and the shadowing effect of the mandible and hyoid bone are visualized. The copyright for this ultrasound picture belongs to the author of this article, Khalid Al-hammuri [5].

Figure 2 shows the view of the tongue contour in the sagittal plane during the image acquisition using ultrasound. The final image of the tongue contour is presented on the ultrasound screen as a bright white concave arc. However, the ultrasound system can detect the tongue image but acoustic imaging is noisy by nature due to the low signal-to-noise ratio, and in the case of rapid tongue movements, there might be missing tongue parts in the image.

**Figure 3:**
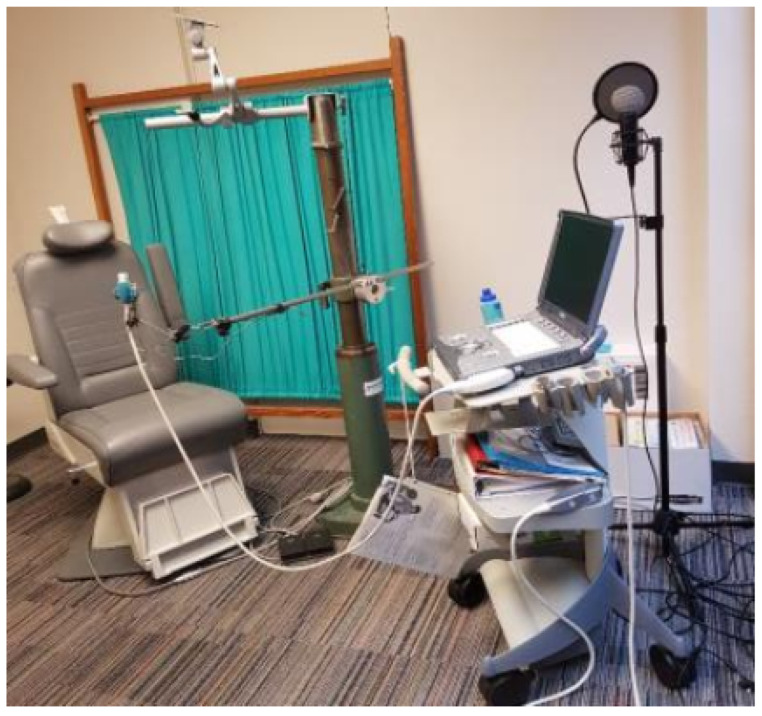
Ultrasound image acquisition system used in speech analysis. The system is also configured with a microphone and head-transducer stability system. The copyright for the ultrasound and head-transducer support system picture belongs to the author of this article, Khalid Al-hammuri [5].

Figure 3 depicts the typical ultrasound system configured with a microphone and the head-transducer support system arrangement [5]. Most of the image acquisition missing data are caused by ultrasound probe misalignment, losing the contact between the transducer and the skin, and the lack of acoustic gel that matches the impedance between the chin-transducer tip [87]. To alleviate image acquisition challenges, different measures must be taken into account. A skilled ultrasound specialist shall conduct the image recording session to properly acquire the image. During the session, it is recommended to use the head-transducer support system to stabilize the head and ultrasound transducer placement to maintain a fixed relative position between the transducer and the head. Furthermore, a convex probe with a small and properly shaped tip area should be used to ensure the ultrasound waveform can pass through the bones to minimize the shadowing effect on the tongue tip and root. In addition, advanced signal and image processing techniques should be used to postprocess and enhance the final image to ensure the data are clean and ready for analysis. In order to further analyze and interpret speech, the system records the sound of the speaker in parallel with the acquisition of the images.

## 3. Evaluation Measures for Tongue Contour Extraction Using Ultrasound

Different techniques are used to evaluate the accuracy of the extracted tongue contour. These techniques use manual or fully automatic extracted tongue contours as reference data. The typical and most accurate methodology to compare the result is by measuring the difference between the segmented tongue contour in the proposed methodology with the extracted ground truth contour. The ground truth data are labelled manually by a human who is specialized in using ultrasound systems. Some researchers use automatically extracted data to validate their results. However, automatically extracted data are less accurate than manual ground truth data. However, they are used when dealing with a massive dataset, as it is time-consuming to produce manual data. Whether the reference data are extracted manually or automatically, the methodology to measure the difference between the extracted and the referenced data is similar and specific measures indicate the accuracy of the methodology. Some measures are valid for either traditional or machine learning techniques, and some other measures are only valid for machine learning techniques.

### 3.1. Mean Sum of Distances (MSD)

The mean sum of distances measure is adopted widely as an evaluation measure for tongue tracking and segmentation; it was proposed by [10]. The mean sum of distances is derived by comparing the automatically extracted tongue contours by the algorithm to the ground-truth-extracted contours by measuring the distances in two main steps. First, the minimum distance between each element on the algorithm-extracted contour and the nearest element on the ground truth is determined. Second, from the ground truth contour, the minimum distance for every point is measured against the nearest point on the algorithm-extracted contour. The sum of the minimum distances from these two steps is divided by the total number of elements in the ground truth and automatically extracted contours to normalize the results. Equation (1) shows the formula for the MSD.

(1) $$MSD\left(U,V\right)=\frac{1}{m+n}\left(\sum_{i=1}^{n}min_{j}\left(|v_{j}-u_{i}|\right)+\sum_{j=1}^{m}min_{i}\left(|u_{i}-v_{j}|\right)\right)$$

where (n) is the contour length of the ground truth, and (m) is the length of the automatically extracted contour, while $\left(v_{j}\right)$ is the manually extracted contour (ground truth) data points, and $\left(u_{i}\right)$ is the automatically extracted contour datasets. On the other hand, $\left(min_{i}\right)$ and $\left(min_{j}\right)$ illustrate the nearest distances between each point on the contour and the nearest point on the other contour, respectively. The MSD has a significant advantage because the length of two contours is not comparable, and other comparison methods such as the mean sum of errors and norm are inappropriate. The MSD is measured in pixels and then converted to millimetres by assuming that each pixel is 0.295 mm [4,5].

### 3.2. Shape-Based Evaluation

Tongue contour image segmentation techniques are evaluated by the shape-based triangle measure proposed by [88]. Equation (2) is used to measure the curvature, while Equation (3) describes the asymmetry of the tongue contour.

(2) $$K=\frac{\left||CD|\right|}{\left||AB|\right|}$$

(3) $$V=\frac{\left||AD|\right|}{\left||DB|\right|}$$

**Figure 4:**
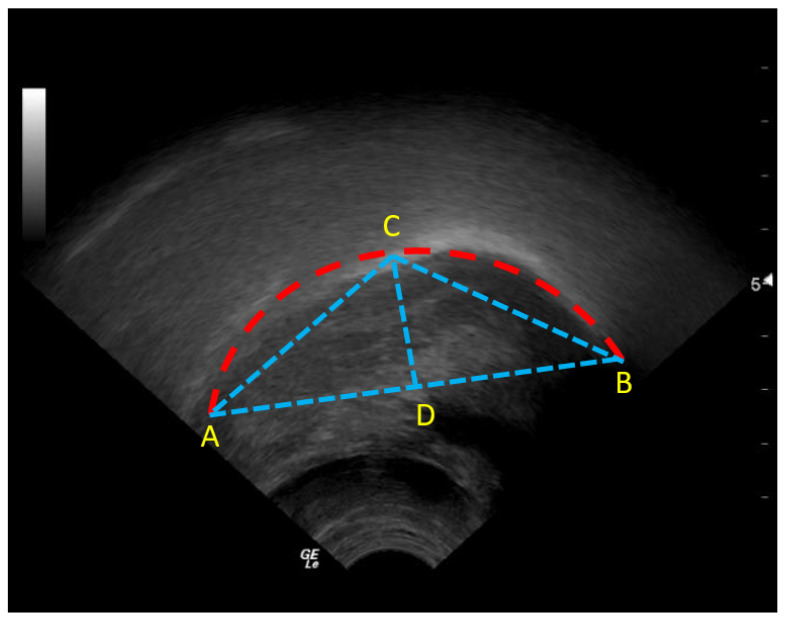
Shape-based evaluation measure. Point (**A**) is on the dorsal tongue part, point (**B**) is the point on the tongue tip, point (**C**) is the apex. Point (**D**) is the projection of point (**C**) on the (**AB**) line. The copyright for this ultrasound picture belongs to the author of this article, Khalid Al-hammuri [5].

This evaluation measure considers the asymmetry and curvature of the tongue shape. ||CD||, ||AB||, ||AD||, and ||DB|| depict the segment lengths that are shown in Figure 4.

### 3.3. K-Fold Cross-Validation

**Figure 5:**
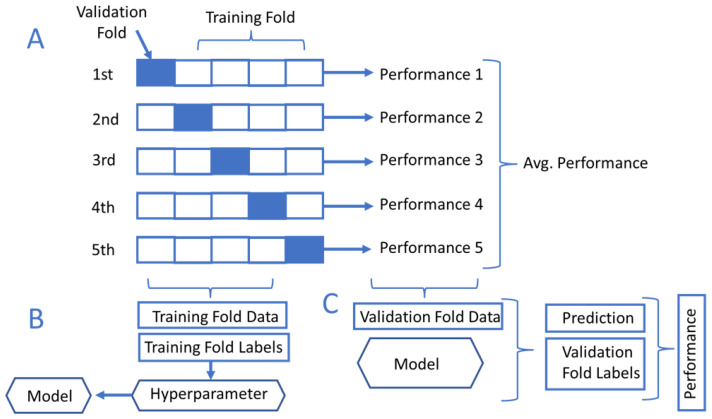
*K*-fold cross-validation process. (**A**) The *K* iterations of the cross-validation. (**B**) The training fold data and labels. (**C**) Evaluating model performance during the validation fold data stage.

Figure 5 shows the data validation on different folds or segments to maximize the model performance. The *K*-fold cross-validation method can be used to evaluate machine learning models’ performance by comparing the training and validation datasets [89]. The *K*-fold process can be done by partitioning the complete datasets into a number *K* of segments. For instance, the typical practice of model validation uses 80% of the segments for data training and 20% for validating the data. The *K*-fold cross-validation shuffles between the *K* segments to reassign different subsets into the validation and training segments. The final performance is evaluated by computing the mean sum of the *K*-folds.

### 3.4. Dice Score Coefficient (DC)

Dice’s similarity coefficient is one of the most important measures to evaluate image segmentation techniques, especially in deep learning algorithms. The Dice coefficient is a statistical tool measuring the similarity between two data sets. The coefficient is important especially in computer vision applications as it can compare the segmented object to the ground truth data and give a sense of how accurate the algorithm is. Equation (4) shows the Dice score similarity coefficient formula.

(4) $$Dice=2x(\frac{U}{A})$$

where (U) is the intersection area between two objects and (A) is the total area of two objects.

#### Mean Square Error (MSE)

The mean square error is the averaged squared error of the datasets. It is a typical evaluation metric to evaluate how accurate the predicted data are compared to the reference data. Equation (5) describes the mean square error mathematical formula.

(5) $$MSE=\frac{1}{n}\left(\sum_{i=1}^{n}{\left(x_{i}-y_{i}\right)}^{2}\right)$$

where (x) is the predicted value, (y) is the observed value, and (n) is the number of data points.

## 4. Tongue Contour Tracking Techniques in Ultrasound Images

This section is a review of the tongue contour tracking methodologies in ultrasound images. There are two main subsections that categorize the tracking algorithms: first, traditional image analysis techniques for tongue contour tracking that review the nontraining-based algorithms, which use a snake algorithm and a graph-based image analysis as core methodologies; second, machine learning-based techniques for tongue contour tracking to review the training-based algorithms that use machine and deep learning.

### 4.1. Traditional Image Analysis Techniques for Tongue Contour Tracking

Tongue tracking by ultrasound was addressed in early research by the cited works [90,91]. However, the process was manual and required a cautious user attention while handling the ultrasound transducer. To enhance the transducer guidance, metal pellets were used as a strong reflector to identify few landmarks on the tongue surface. The landmarks were used as a reference to monitor tongue movement during swallowing by comparing the pellets placed on the tongue anterior and posterior segments to the hyoid bone reference at different stages of movement.

There are two main traditional methodologies used to segment the tongue: active contour model (snake algorithm)and shape consistency and graph-based tongue tracking models.

#### 4.1.1. Active-Contour-Based Methodologies (Snake Algorithm)

To automate tongue contour tracking, many researchers have relied on the snake algorithm [92,93] as the base algorithm for most of the traditional techniques in tongue contour tracking. The snake algorithm is an active contour and energy-based method that adapts to get closer and closer to the object until reaching a certain threshold or energy constraints to fit the object boundary. The snake algorithm has been used widely in vision tasks such as the detection of lines, objects and subjective contours, and motion tracking. In the case of lingual ultrasound, the snake algorithm can be useful for interactively segmenting a tongue contour by applying certain user-imposed constraint forces to localize the tongue features of interest. Examples of the first attempts to use active contours for tongue tracking tasks were provided by [94,95,96], which were made by the same authors and improved consequently.

An adaptive snake algorithm was introduced by [94]. The authors collected 2D ultrasound images and used a head and transducer support system to stabilize the ultrasound transducer. In the first frame, a human expert selected a few candidates of the contour points to generate the initial tongue contour to initiate the snake algorithm. For the following frames, the researchers proposed an adaptive model that estimated an optimized contour that matched the tongue contour edges on each frame. Finally, the algorithm implemented a postprocessing technique to enhance and refine the extracted contours.

The cited work in [95] followed the same process as the work in [94] and extended the work using different constraints to test it in speech and swallowing applications. The authors in [95] showed an improvement in the model performance by minimizing the computational cost to make it more flexible for a variety of different tasks.

Similarly, the algorithm proposed by [96] required an initial input from an expert to delineate the tongue contour on the first image frame to ease the snake algorithm optimization of the energy constraints that enforced the detection of tongue contour edges in the desired region of interest. Subsequent video frames were processed by adapting the initial contour edges to match the tongue deformation. External and internal energy functions were suggested to optimize the tongue contour’s external edges and concavity, respectively. Although the methodology showed some success in tongue contour detection, its performance dropped drastically in the case of noisy images due to its sensitivity to speckle noise. Moreover, in the case of rapid tongue movements, the external energy function could fail to adapt the edges and match the tongue boundaries’ deformation to the new position at the next frame. This, unfortunately, limited the ability of this methodology in real-time processing as it could fail suddenly during the video processing in real time.

Publicly available software EdgeTrack [2] proposed an improvement to the mentioned work in [96]. EdgeTrack implemented an enhanced methodology for the active contours that incorporated the gradient, local image information, and object orientation, unlike the classical methods that relied only on the gradient information [2]. This improvement optimized the contour’s lower boundaries and rejected any undesirable edges unrelated to the tongue. EdgeTrack software had a few technical limitations, and like any other deformable models, it could misidentify the true tongue contour’s edges. EdgeTrack did not have any preprocessing capability, reducing the snake algorithm’s efficiency as it is sensitive to noise. The software program could not process a long video sequence with more than 80 frames, limiting it to short recordings. This is not beneficial in the case of long speech processing sessions or a real-time analysis. EdgeTrack was computationally expensive because the algorithm relied on complex optimization techniques. In some cases, when there was a rapid movement during the speech, the tongue contour had a visible deformation that looked like a concave arc; the software tool failed because it did not use temporal smoothness in the minimized internal energy function. EdgeTrack results were validated by two experts who delineated the tongue contour manually. The mean sum of distances (MSD) accuracy measure was used to compare the results between EdgeTrack and manual ground truth data. The reported results were in the range of 1.83–3.59 mm for the MSD.

The multihypothesis approach [4] combined the traditional motion model, snake algorithm, and particle filter to track the tongue contour. The first step toward building the algorithm was by deriving a motion model based on manually prelabelled images. Next, tongue contours were extracted and then normalized with respect to the length and position. Following that, a principal component analysis (PCA) and mean shape were estimated, then the covariance matrix was computed by using the information from the tongue motion information such as the scale, shape, and position.

The snake algorithm used in [4] required to be initialized to process the tongue tracker by manually identifying points on the contour at the first frame to segment the tongue. After that, the particle filter was created by copying the segmented contour for a defined number of so-called particles. Next, a multihypothesis approach was created from each copied particle of the previous frame based on the derived motion model of the tongue scale, position, and coarse shape. The derived tongue contour model was then adapted using the snake algorithm to fit the tongue contour accurately. A band of energy-optimized constraints was used to choose the best particle by ensuring that the tongue contour was below the bright white arc on the tongue’s upper surface. Two groups of subjects with Steinert’s disease (a form of myotonic dystrophy that causes slow speech, distorted vowels, and consonants) and healthy subjects were used to validate the research study. The reported accuracy was 1.69 ± 1.10 mm for the mean sum of distances (MSD). However, the approach claimed that it was not highly dependent on the training data. The segmentation accuracy was still dependent on the number of particles, which increased the snake algorithm’s computational complexity [4].

To fully automate the tongue contour extraction without using training data or human interaction, some researchers designed multistage techniques [6]. Unlike other semiautomated methodologies such as those in [2,3,97], which required human interaction in the first frame, this methodology initiated the active contour model by automatically deriving candidate points on the tongue contour. These points were identified by applying the phase symmetry method for image enhancement. Then, the image was skeletonized, and data points were clustered to select the best candidate points. These candidates were used as initialization points for the algorithm. The accuracy improved by implementing two methodologies for algorithm resetting or reinitialization in a frequent and timely manner order. According to the results, the measured mean sum of distances (MSD) accuracy measure was similar to that of other semiautomated techniques. They claimed that the MSD was 1.01 mm and 0.63 mm for their fully automated and reinitialized techniques, respectively. The reported results were highly accurate with some frames, but this may not be easy to achieve when processing videos in real time.

However, relying on the active contour model for tongue tracking in ultrasound images is error-prone and maybe not the most efficient technique. In some cases, it can lead to ultimate failure due to the number of constraints needed for the model adaption, which is difficult to predict for all cases accurately. Although the approach in [6] proposed a novel methodology for automating the process of identifying the active contour initialization and reinitialization parameters, this was still not enough to produce highly accurate results in a global and generalized context. There are many variations in ultrasound imaging modalities that produce different imaging qualities, making it difficult to track the tongue contour using the same active contour model constraints.

The similarity-constrained active-contour-based methodology for tongue tracking proposed in [98] suggested a technique that coped with the tongue contour tracking errors and missing data based on the tongue shape from previous contours to minimize the effect of missing data. In order to deal with the accumulated error during the continuous tracking of the tongue contour over a video sequence, a complex-wavelet image similarity index (CW-SSIM) was proposed to reinitialize the tongue tracker automatically. This algorithm showed an advancement compared to traditional techniques by handling missing data and using an automatic reinitialization. However, it was still based on the active contour, which is error-prone and sensitive to noise. Too many constraints would enhance the model accuracy but increase the computation cost. The best-reported results using similarity constraint + CW-SSIM were an MSD of 0.9912 ± 0.2537 mm.

As mentioned before, all methodologies that are based on the active contour may suddenly fail and the tongue tracker would stop. An initializer, either manual or automatic, is needed to enhance the accuracy of tongue tracking. The researchers in [99] conducted a comparative study on the effect of an automatic reinitialization technique to enhance the well-known traditional image segmentation. The automatic reinitialization enhanced the results from an MSD of 5–6 pixels to about 4 pixels (1 pixel = 0.295 mm). The MSD accuracy results without the need for automatic reinitialization for the well-known tongue tracking tools EdgeTrack and TongueTrack were 7.06 ± 2.77 pixels and 5.59 ± 3.04 pixels, respectively. The MSD accuracy after using the automatic reinitialization was 3.46 ± 1.04 pixels and 3.60 ± 0.96 pixels for EdgeTrack and TongueTrack, respectively.

#### 4.1.2. Shape Consistency and Graph-Based Tongue Tracking Methodologies

Researchers derived an active appearance model to predict the tongue contour shape on ultrasound images in [100]. The active appearance model was inspired and estimated using a manual delineation and extraction of the tongue contour from tongue X-ray images. The results were compared to those of EdgeTrack [2] and the constrained snake algorithm [101], which combined ultrasound, EMA, and recorded voice to predict the tongue shape. The work in [100] showed an improvement in root mean square error compared to that of [2,101]. The active shape model (ASM) was also evaluated and used in [91]; the authors showed that the ASM was efficient and powerful for phonological applications. It was able to capture the tongue motion variation by capturing the temporal information. It was also useful for either automated or semiautomated techniques.

Lingual ultrasound tracking was introduced in another well-known software called [3] TongueTrack, which could process a sequence of 500 frames. The methodology considered contextual information and advanced optimization techniques to estimate unpredictable tongue motion. The reported accuracy was 3 mm, making it acceptable for segmentation purposes. The tool used a higher-order Markov random field energy minimization framework. The results were validated with the ground truth data from two different groups of 63 acoustic videos [3].

The process of TongueTrack required an initial human interaction by manually delineating a few points on the first tongue contour to be used as an initializer for the algorithm. After that, the delineated points were fitted by using a curve-fitting polynomial function to build a continuous and smooth contour. Next, a solution-space label set was created by generating an estimation model for the dynamic tongue motion. This label set was used to compare each contour with the minimized Markov random field energy module in each subsequent frame. It processed it iteratively until reaching a predefined threshold; it was predefined as 2 mm in [3]. The tool obtained good results, but it had a few drawbacks. The software tool could not process long video frames. At the same time, the algorithm optimizer might not converge properly, leading to a sudden failure in tracking progress as it required 20 iterations to optimize nine parameters. Moreover, the algorithm needed a manual reinitialization by delineating the tongue contour by hand, limiting its efficiency for real-time processing.

Tongue contours are also tracked in ultrasound images by using graph-based analysis of the temporal and spatial information during speech [102]. Spatial information is essential to extract tongue features from each image on a single frame. At the same time, the temporal resolution is necessary to predict the intrarelationship between the entire sequence of image frames extracted from the video session of the speech. The tongue tracker was implemented as an optimization problem using a Markov random field energy minimization. The algorithm enforced temporal and spatial regularization constraints to ensure tongue tracking reliability.

In the landmark-based tongue contour tracking [97], the tongue shape was predicted based on the position of a few pellet plates used as landmarks on the tongue surface. The landmarks were extracted from the available articulatory database. The available landmark positions were smoothed using the spline function and compared to the ground truth data extracted by ultrasound images. Tongue contours extracted by ultrasound helped to identify the optimum number of required landmarks to get the desired accuracy of 0.2–0.3 mm for any future use.

Another research study coped with the tongue tracking problem by modelling it as a biomechanical method [103]. The methodology was initialized by manually drawing a closed contour around the external and internal edges of the tongue. The Harris feature detector was used to identify the one hundred most significant corners or edge features. The detected points were sorted in descending order based on the quality of the feature. An optical flow algorithm was then used to estimate each point’s displacement in the consequent frames. The corner feature displacement estimation was approximated only in the neighbour pixels (around 15–20 pixels) to minimize the displacement error in case of any missing data. In order to minimize the uncertainty of the estimated features, a covariance matrix was computed. The accuracy was measured by the mean sum accuracy, which was reported between 0.62 mm and 0.97 mm. However, the study faced many challenges. The algorithm required many parameters and constraints to be computed in order to estimate the displacement. Relying on the Harris feature detector may not have been efficient, especially in the case of rapid tongue movement, missing details, or extreme deformation, as it was almost impossible to guarantee that the same detected corner features were visible in the next frame within the neighbourhood pixel constrains.

An interactive approach for lingual ultrasound segmentation that incorporated four stages from preprocessing to the segmentation and postprocessing analysis was introduced in [5]. In the first stage, and unlike other methodologies that ignored an essential part of image denoising, the thesis implemented novel denoising techniques by using a combined curvelet transform and shock filter. In the second stage, the thesis derived an interactive model that predicted the tongue area of interest to minimize the computation complexity and contour tracking error. The third stage focused on tongue contour extraction and smoothness. The fourth stage proposed a new technique that transformed the extracted tongue contour from an image state to a continuous signal which resembled a full video for all frames. The advantage of this technique was that it enabled the researcher to extract a unique signature of each sound; this could be beneficial for training a machine learning model on sound pattern recognition. The tongue contour segmentation results were validated and compared to ground truth data. The mean sum of distances (MSD) was 0.955 mm.

### 4.2. Machine-Learning-Based Techniques for Tongue Contour Tracking

**Figure 6:**
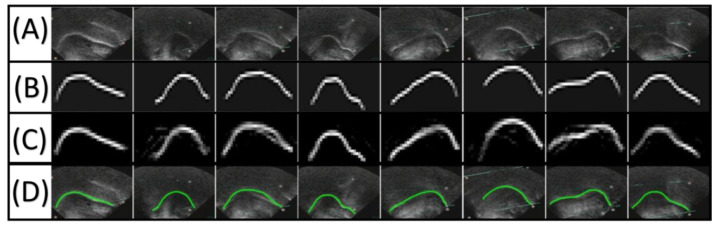
The process of labelling ultrasound images and extracting tongue contour using a deep belief neural network. All labels from (**A**–**D**) are horizontally ordered. (**A**) Ultrasound image before processing. (**B**) Manually labelled ground truth data. (**C**) Extracted features from ultrasound images using a translational deep belief neural network. (**A**) Extracted tongue contour overlaid on the original ultrasound image [104].

One of the early attempts to use deep learning for automatic tongue extraction was made by [104]. Their methodology, Autotrace, was implemented using a translational deep belief neural network (tDBN), which was based on restricted Boltzmann machines (RBMs). The network was trained based on human-labelled and generated sensor data. The hybrid data training methodology was efficient for improving tongue contour segmentation accuracy. However, there were discrepancies in the segmentation of some image frames and model-segmented tongue-unrelated parts. The results were validated by using a five-fold cross-validation, and the reported accuracy was measured by an average mean sum of distances (MSD) of 2.5443 ± 0.056 pixels (1 pixel = 0.295 mm [2]). The algorithm segmentation capabilities were fair enough; however, a postprocessing algorithm was needed to refine and enhance the final tongue contour segmentation. Figure 6 depicts the ultrasound image, manually labelled tongue contours and the extracted tongue contours proposed by [104].

To improve Autotrace [104], researchers in [105] proposed a new technique that automatically labelled the tongue contour, followed by training the algorithm in two phases. Using a deep autoencoder, the algorithm learned the relationship between the extracted contour and the original ultrasound image. By using the training data, the algorithm was able to reconstruct the tongue contour from ultrasound images without human intervention. The results were validated by comparing the average mean sum of distances between the hand-labelled and the deep-learning-extracted contours. The average MSD was reported as 1.0 mm, making it applicable to lingual ultrasound applications.

Based on the principal component analysis (PCA) and a neural network, an automatic algorithm was designed to segment the tongue contour [106]. The PCA-based feature extractor, Eigen Tongue, was used to extract the tongue contour features from the ultrasound images. The visual features of the extracted Eigen Tongue were processed using an artificial neural network based on the PCA feature model. The model was evaluated by using 80 annotated images from nine speakers. The average error measured by the MSD was reported to be around 1.3 mm.

Typical convolutional neural networks were used to classify the tongue gesture from B-mode ultrasound images on the midsagittal plane in [107]. The researchers used data augmentation to increase the size and versatility of the data, which increased the algorithm’s performance. The reported accuracy results for the classification task were 76.1%. Further improvements were suggested as future work. The recommended improvements were in the model optimization or combining the methodology with a hybrid technique such as the ensemble method.

The well-known U-net architecture [108] was used by [109] to automatically extract the tongue contour in ultrasound images. The algorithm was trained by using 8881 human-labelled images collected from three subjects. The results were validated by using the Dice score, which was 0.71. Relying on the Dice score only for validation is not enough. More validation is needed for their methodology, such as the mean sum of distances (MSD) measure, which has become a de facto standard in the lingual ultrasound accuracy measures. The MSD provides a reliable measure that considers the variation of the tongue contour length, which normalizes the sum of distances over the tongue contour length. To further enhance the performance, it might be needed to use a hybrid technique and larger dataset.

To automate tongue segmentation, a convolutional-neural-network-based architecture was utilized in [110]. They compared the efficiency of using the U-net [108] and Dense U-net [111] architectures to extract the tongue contour. These architectures have become de facto models of biomedical image segmentation and gained a wide popularity in the field. The results showed that Dense U-net was more generalizable for a wide variety of datasets. At the same time, the standard U-net architecture could perform the tongue extraction task faster. After extracting the tongue contour, it had to be postprocessed. In the postprocessing stage, the output was fed into a probability heat-map model, where the intensity of each pixel corresponded to the probability of each part of the tongue [110]. A 50% threshold was applied to filter out any undesired predictions. The remaining output was skeletonized to reduce the segment thickness. Following that, the results were smoothed and interpolated using the UnivariateSpline function in the SciPy package in Python. The final output was a hundred points to represent the predicted tongue. The algorithms were evaluated using the MSD for the 17,580-frame dataset. The reported MSD results for the 32×32 data size were 5.81 mm and 5.6 mm for U-net and Dense U-net, respectively. The research also showed that data augmentation and the loss function significantly affected model performance other than stacking more layers.

Two deep learning architectures were designed, BowNet and wBowNet, to extract the tongue contour from ultrasound in [112]. With the integrated multiscale contextual information, the decoding–encoding model had the ability for global prediction. The dilated convolution had the local searching capability of preserving image features more than standard convolution, making it valuable for medical imaging applications to retain fine image details. The two architectures enhanced the final prediction results by combining the local and global searching. The mean sum of distances for BowNet and wBowNet compared to the greyscale ground truth images was in a range of 0.2874–0.4014 in pixels for BowNet and 0.1803–0.3588 pixels for wBowNet. However, the reported results appeared to be almost perfect, which is not easy to achieve in the case of a complex analysis of lingual ultrasound. The researchers need to provide more information about the data validation in a generalized clinical context by using a dataset from a different source.

A simple approach to extracting the tongue contour by training a deep network on landmarks annotated on the tongue contour was developed in [113]. These landmarks were automatically and randomly selected on different points by using annotation software. The model architecture was called TongueNet, and the results were validated by the mean sum of distances which achieved 4.87 pixels.

Using U-net and the lighter version of sU-net in a thesis work, a deep learning approach was implemented to segment tongue contours [114]. In their thesis, the researcher emphasized the validity and performance of deep learning models to segment the tongue contours from ultrasound images. However, they suggested that the deep learning model they used only focus on the spatial information on a single image frame without considering the temporal information that handled the full speech in the video sequence. The thesis [114] also discussed the limitations of their deep learning model in their generalization capability of feature extraction, as they inherited the nongeneralization of convolutional neural networks (CNN) models, which is the core of a deep learning model such as the U-net architecture. The thesis suggested using data augmentation to enhance the model training by considering the variation and image transformation to handle different cases at different scales.

A denoising convolution autoencoder (DCAN) model to process B-mode ultrasound images was investigated in [115]. The model reported being able to extract image features due to its ability to denoise and retain the resolution of the reconstructed input from the ultrasound. It was tested on reconstructing ultrasound images in speech-related applications. The research compared the DCAN to other three well-known autoencoder architectures, the deep autoencoder (AE), the denoising autoencoder (DAE), and the convolutional autoencoder (CAE). The reported result showed that the DCAN had a 6.17% error rate in identifying words in a silent-speech recording test [115].

Researchers implemented a novel technique that harnessed the spatial–temporal analysis to predict future tongue movement based on a short recording of the past tongue motion in [116]. The research used a combination between a convolutional neural network (CNN) and long short-term memory (LSTM), which was called ConvLSTM. The advantage of this combination was that the CNN had the ability to segment tongue contour in each image frame to extract spatial information. However, it could not process the temporal information of ultrasound image sequence frames. On the other hand, LSTM was used in processing data sequence in one dimension, making it efficient for temporal information data prediction, but at the same time, it was unable to handle images in two dimensions (2D). The ConvLSTM could handle image data in 2D and predict future data based on the history of tongue motion. The ConvLSTM results outperformed the three-dimensional convolutional neural network (3DCNN) in predicting future tongue contours. The ConvLSTM was able to predict the future nine frames based on data from the previous eight frames. We believe this algorithm was not only important for data prediction of tongue contours, but it might be helpful for generating more data that are close to real data to train larger deep learning algorithms such as a Transformer model or a graph neural network.

An algorithm combining an image-based segmentation model, U-net, and a shape consistency regularizer was proposed by [117]. The combination provided a solution to the missing data in ultrasound images by predicting the information based on the consideration of the sequential information of the shape regularizer. The regularizer was derived based on the similarity between adjacent image frames. The results were validated by computing the MSD of the tongue contour data segmented by the U-net algorithm using different loss functions. The quantitative validation showed that the combination between the regularizer and cross-entropy loss (CE) obtained the best results among the other compared losses such as the Dice coefficient (DC) or the active contour loss (AC). The CE+regularizer reported having an MSD of 2.243 ± 0.026 mm.

To improve the well-known U-net architecture, researchers proposed a tongue contour segmentation algorithm called wUnet [118]. The main modification of wUnet was replacing the skip connection in typical U-net with a VGG19 block. The researchers claimed that the new algorithm surpasses U-net by passing more information to the decoder to compensate for the information loss during the convolution within the encoder. The wUnet validation results showed an MSD of 1.18 mm compared to 2.26 mm in the U-net architecture.

A system based on a deep learning technique was designed to predict silent speech using ultrasound images in [119]. The system was trained on audio features recorded synchronously with ultrasound images using a deep convolutional neural network. The system was designed to predict the speech sound from the silent speech based on the training data. This methodology could be beneficial for human–machine interaction in smart devices.

To update an older silent-speech benchmark study [74], the work [73] used a deep learning approach for the same benchmark. The new study used a deep autoencoder to train the collected dataset from acoustic tongue and lips movement videos, which were collected at the same time.

The research [9] used ultrasound videos to extract tongue features using deep learning. The dataset was collected from 82 speakers and trained using the Kaldi speech recognition toolkit [120]. In terms of speech analysis, the research suggested two methodologies. The first one was the utterance or speech duration, which was measured based on the syllable rate. The second one was the articulatory area, which was measured by estimating the convex hull area, which was the area under the tongue contour spline that formed a convex-like shape when extracted from the ultrasound images using the MTracker tool [109]. Following that, a postprocessing was performed by the isolation forest method [121]. The research found that the silent articulation exhibited a longer time compared to the model speech.

## 5. Results and Discussion

Qualitative and quantitative evaluations were used to evaluate the performance of the tongue segmentation from ultrasound images. Traditional and machine learning algorithms have different abilities for tongue image recognition to make each methodology unique on its own. In the qualitative analysis, we propose a qualitative scoring matrix that considers the final image quality, shape consistency, and algorithm complexity to test the method’s usability performance. In the quantitative evaluation, we consider the MSD as a primary measure and some other measures such as the RMSE, MSE, and word error rate as secondary measures for some other applications.

### 5.1. Qualitative Evaluation

Among the traditional techniques that are based on the snake algorithm, the multihypothesis approach [4] produces robust research to handle tongue tracking efficiently. The output image quality is acceptable for speech recognition tasks. However, the quality of the image depends on the number of particle filters that are used, which makes this technique not practical for real-time applications. The algorithm is also tuned based on the tongue shape and motion model derived from different image frames. There is a trade-off for using a motion model. It may help to increase the confidence ratio of the segmented tongue contour. However, at the same time, the derived motion model may be inaccurate and cannot be applied in a general perspective. The research in [4] has some limitations that can be addressed efficiently using deep learning algorithms based on an attention mechanism such as Transformer.

Publicly available tools such as EdgeTrack [2] and TongueTrack [3] are inefficient in real-time processing. They are susceptible to sudden and frequent failure during the segmentation and require a manual reinitialization to continue the processing. The image quality for their segmented contour is fair but is not suitable for medical-grade applications. These algorithms could not address the missing data issue and the variation of the shape consistency. The main drawback of these algorithms comes from the heavy optimization of too many parameters. The optimization issue does not just make them slow but also very limited to a specific subset of data and they cannot be applied for real challenges outside the lab. TongueTrack has an advantage over EdgeTrack by considering the spatial information between different frames. We believe if they used image denoising and a region-of-interest selection, the burden of computation complexity could be minimized. For future work suggestions, using a U-net architecture could be efficient for removing image noise and extracting image features, then combining them with existing algorithms as a hybrid technique.

The biomechanical method [103] derived a motion model for the tongue contour geometrical movement based on previously labelled X-ray images. The motion model alongside a Harris feature extractor were used to track the tongue features. The Harris feature extractor has too many limitations because it is sensitive to noise and requires localization constraints to select tongue contour features around the desired region of interest. In real-time tracking techniques, it may not be accurate since tongue motion may be more significant than the suggested local constraints. The final image and the extracted contour are susceptible to a high degree of uncertainty, making it not efficient for prediction using the suggested pipeline. The idea of using X-ray images to extract the motion model is good if we consider image quality compared to ultrasound. However, it could be risky to train the data from data with different distributions or statistical characteristics, requiring additional analysis. In future work, we recommend using deep learning algorithms instead of unrealistic motion models to merge ultrasound and X-ray images. Image fusion with deep learning models could be a potential solution for this problem as they can merge the quality of X-ray and ultrasound images using some image features or landmarks.

On the other hand, [5,6], unlike most traditional techniques, implemented denoising techniques to enhance the image and refine the tracking accuracy. However, the paper [6] relied on the snake algorithm as a base algorithm but with an automated reinitialization technique. The automatic reinitialization technique was robust enough to handle the sudden failure of the active contour. It might be more efficient than EdgeTrack and TongueTrack. However, the algorithm [6] still relied on too many constraints to optimize the snake algorithm. As mentioned before, this limits the ability to predict and estimate tongue displacement in a global context, making it unrealistic to predict the performance of any new data from a new source. In comparison, the research proposed in [5] went in a different direction to track the tongue without using the snake algorithm. A combined curvelet and shock filter denoised the image, then based on the temporal information of previous contours, an adaptive tongue region of interest was implemented. To extract a unique signature of each speaker, the tongue feature was extracted and transformed into speech time series data. In future research, we recommend combining the algorithm proposed in [5] with deep learning. The proposed research in [5] was robust for feature extraction using a policy-based adaptive model to extract features but had some limitations for real-time applications. Similarly, we recommend the algorithm [6] as a postprocessing tool combined with deep learning in a hybrid tongue contour extraction and refinement technique.

In deep learning methodologies, the research on convolutional neural networks to automate tongue segmentation [110] used the de facto segmentation models in biomedical imaging analysis, U-net and Dense U-net. Dense U-net had more generalization capability, meaning it could extract more features in a global context. It would be more accurate for any dataset outside the training set. However, Dense U-net is slower than the traditional U-net architecture which makes traditional U-net more efficient in real-time segmentation. Autotrace [104] used a translational deep belief network for image segmentation and was improved by [105] using a deep autoencoder. The deep autoencoder relied on the user data input, which affected the results for a limited context of given data. BowNet and wBowNet [112] and TongueNet [113] suggested two techniques for the tongue segmentation task based on multiscale contextual information and a deep network of landmarks. In general, most deep learning algorithms are based on CNNs, which is helpful for feature extraction and noise removal in a local context. However, the intrarelationship between the sequential image frames is limited. We suggest combining a CNN and any other deep learning-based spatial–temporal analysis to process continuous data. Some of the suggested algorithms are Vision Transformer, Vision-Graph, and ConvLSTM.

The authors in [116] proposed a ConvLSTM architecture. ConvLSTM is a novel approach that derives temporal information from the ultrasound images by extracting the intraframe relationship to resolve the issue of the lack of temporal resolution of other techniques. The model could predict tongue shape in the consecutive nine frames based on the data from the previous eight frames. In the same manner, [117] proposed a tongue contour tracking algorithm using a state-of-the-art U-net architecture alongside a temporal shape-consistency-based regularizer. This methodology was one of the most reliable techniques for real-time tongue processing. In their method, they used it to predict future frames, which could be used for training larger and more efficient algorithms such as the Transformer model. The Transformer model is gaining popularity as the state-of-the-art algorithm in the field due to its performance and predictability. The Transformer model also has some limitations, and it needs a huge dataset for training; this could be alleviated using the transfer learning methodology. Moreover, Transformer requires a fixed size of the input. LSTM also has limited memory but does not need a huge dataset like Transformer. The final suggestion is to use attention-based algorithms such as the Transformer model if the dataset is huge. If the dataset is small, LSTM can be used. Regarding image quality for deep learning, U-net is well known for preserving image features and noise removal. At the same time, attention-based algorithms are robust for predicting the correct speech behaviour to produce a high-quality output.

**Figure 7:**
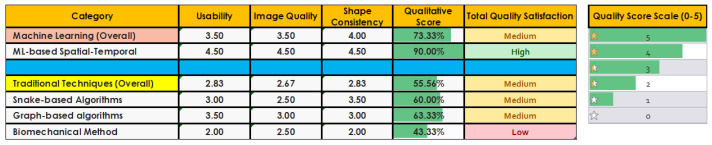
Quality evaluation matrix. Usability, image quality, and shape consistency are scored on a 0–5 scale (0 is the lowest and 5 is the highest). The final quality score is shown on a percentile scale and a satisfaction rate from low to high.

**Figure 8:**
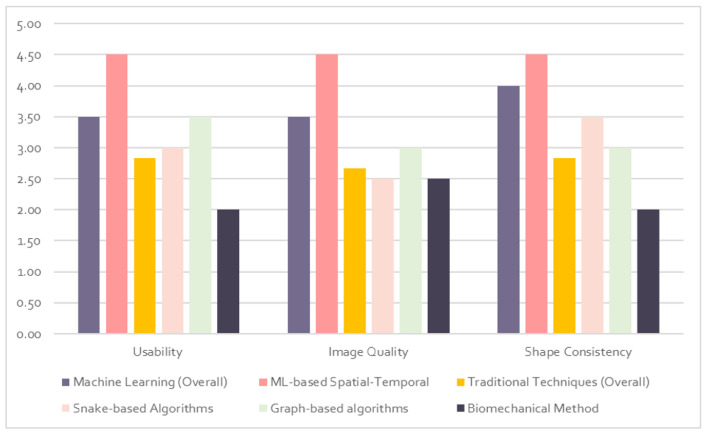
Bar chart for the total qualitative score of tongue image segmentation categories. The Y-axis is the qualitative score probability, and the X-axis is the quality score category for each image segmentation technique.

Figure 7 and Figure 8 depict the quality evaluation matrix and bar chart for the total qualitative score of each category of tongue segmentation techniques. Image quality is generic and difficult to measure. Due to the lack of a definitive standard for image quality, we are proposing a new matrix that scores image quality based on different factors. In order to determine the image quality, we use the visual inspection and structural similarity index measure [122,123]. In the usability measure, we mainly consider the algorithms’ generalization and scalability. A generalized algorithm is one that performs well in real-life situations as well as in lab testing. For the scalability measure, we define an algorithm as scalable if it is not sensitive to the variation in use-case environments or data size. This is crucial to ensure the algorithm is viable for use in different scenarios, not just optimized for one solution. The consistency of shape is essential to determine whether the predicted shape is actually a tongue or not. We measure the shape consistency by comparing the results with ground-truth-labelled images and the data collected from different algorithms. The qualitative evaluation matrix is scored on a 0–5 scale (zero is the lowest and five is the highest). The final quality score is depicted on a percentile scale and evaluated with a satisfaction rate from low to high.

### 5.2. Quantitative Evaluation

The primary quantitative measure to evaluate tongue contour segmentation in this article was the MSD. The MSD is valid for this problem as it uses averaged measures to account for the tongue contour variation. The average MSD for the machine learning approaches was 1.4 mm, and the average MSD for the traditional techniques was 1.65 mm. The accuracy of these measures can be arguable as it is difficult to judge these results in realistic applications. These methods are never used in production and never tested outside the lab. One of the common challenges in image recognition or machine learning is when the designed models typically fail when used outside the lab while they pass the testing stage in the lab. Poor performance may result from a small training dataset or an insufficiently generalized model (a generalized model performs well in testing and training). To transfer the model from research to the successful production stage, we recommend using a cloud-based solution to scale the designed model and evaluate the performance in different environments. In order to increase dataset diversity, we recommend data augmentation techniques. Moreover, transfer learning could be a viable solution if limited data are available. Transfer learning is using features from pretrained models such as Imagenet [124] or VGG19 [125] and then fine-tuning the algorithm on the target datasets of the tongue images. Transfer learning minimizes the training time and enriches the model with low-level features such as edges and textures to help with data size limitation and to obtain more statistically accurate results. On the other hand, data augmentation helps to generate new data. Data augmentation can be simple, such as transforming data, rotating it, and flipping it, or more complex, such as creating new images using generative adversarial networks (GANs) [126].

There are different validation measures considered in addition to the MSD. Some of these measures are RMSE, MSE, speech recognition success ratio, word error rate, mean segmentation error, and accuracy. The fact is that there is no definitive recipe for the validation, and a combination of different measures is needed to address each methodology.

The MSD is considered a reasonable measure compared to the RMSE and MSE. For instance, the RMSE is helpful in regression analysis when we want to consider lower residual values unlike the MSE, which is biased towards higher values. The RMSE was used in [97] and the reported result was 0.2–0.3 mm, which was not meaningful statistically to be considered as a reference for tongue segmentation standard. The MSE was reported in [116] and the result was 17.3 mm. The better MSE is, the closer to zero. The problem with this measure is that it is sensitive to outliers or abnormal values, which maximize higher values; this explains why the error was high in [116]. To use the MSE correctly, the researcher should be careful in the feature engineering stage to remove unnecessary data. A logarithmic scale sometimes helps in this case. Accuracy was also used in the biomechanical method [103]; they reported a result of 0.62–0.97 mm. Accuracy is a generic and simple evaluation measure. It has severe limitations in the case of data imbalance and does not account for the variation in data size.

Some other used measures such as speech recognition success ratio which was reported in [119] as 65% for their algorithm evaluation. It only provides a counting measure for the final speech success rate, but not for the tongue segmentation accuracy. It is not valid in the case of data variation, since it neither considers nor accounts for the statistical distribution. The word error rate was also reported in [115]. It can provide a general impression of performance, but it does not provide any meaningful or accurate information about the tongue; it does not provide any clinical measure. The mean segmentation error was used in [102]; their results were reported for dense and sparse data as 4.49 mm and 2.23 mm, respectively. This technique was compared to the MSE, but the researchers enhanced it by adding additional optimization techniques to remove unnecessary data. This is a significant enhancement compared to the MSE evaluations, but it is not as efficient as the MSD, which represent the most reasonable measure that can be valid to evaluate tongue segmentation techniques.

Table 1 compares the most important techniques used to segment tongue contour from ultrasound images by describing each method’s core methodologies, results, data types, and limitations.

## 6. Conclusions

Various methodologies have been employed to extract tongue contours from ultrasound images, with varying degrees of success. There are advantages and disadvantages to each methodology. This paper presented methods for tongue contour segmentation from ultrasound images using two main categories of techniques. The first category was traditional image analysis. The second one was machine learning-based techniques. The traditional techniques relied mainly on active contour (snake algorithm), shape consistency, and graph-based methodologies. Machine-learning-based algorithms used mainly CNN, U-net, and LSTM architectures.

The significance of this review article is to provide the researcher with a comprehensive quantitative and qualitative evaluation of the tongue contour tracking techniques in ultrasound images.

Based on the results, the machine-learning-based algorithms are superior to other techniques considering the segmentation accuracy and the proposed qualitative measure such as usability in real-time application, image quality, and shape consistency. The traditional techniques are robust for feature extraction and postprocessing applications, as they are specifically optimized for the tongue segmentation task.

We conclude that the key to obtaining more accurate results is by using a hybrid combination of machine learning and traditional techniques. Machine learning is efficient as a real-time tongue segmentation tool. On the other hand, the use of traditional algorithms can enhance a machine learning model output by using interactive user segmentation tools during the training and postprocessing stages.

## Figures and Tables

**Table 1:** Comparison between different tongue contour segmentation methodologies.

Method	Category	EV. Measure	EV. Result	Data Type	Core Methodologies	Limitation
EdgeTrack [2]	Traditional	MSD	0.53–1.0 mm	Tongue US images	Snake algorithm + gradient + local image information and object orientation	Sensitive to noise, computation complexity, can process only 80 US frames in one session
TongueTrack [3]	Traditional	MSD	3 mm	Tongue US	Higher-order Markov random field energy minimization framework	Needs manual reinitialization, sensitive to noise, can process only 500 US frames in one session
Tongue shape prediction from landmarks [97]	Traditional	RMSE	0.2–0.3 mm	Tongue US + EMA or X-ray	Spline interpolation + Landmark mapping using metal pellets	Difficult to use due to the limitation of the data collection
Graph-based [102]	Traditional	Mean segmentation error	Dense = 4.49 mm Sparse = 2.23 mm	Tongue US	Image graph-based analysis + adaptive temporal regularization using Markov random field optimization	Computation expensive due to optimizing too many parameters
Biomechanical [103]	Traditional	Accuracy	0.62–0.97 mm	X-ray and US images for tongue and vocal tract	Harris features + optical flow	Sensitive to noise; not practical for ultrasound as it was trained on X-rays
Multihypothesis approach [4]	Traditional and machine learning	MSD	1.69 ± 1.10 mm	Tongue US images	Snake algorithm + particle filter	Computation complexity, needs too many filter parameters to get accurate results
Computer vision-based tongue tracking and feature extraction [5]	Traditional	MSD	0.933 mm	Tongue US images	Image denoising + tongue adaptive localization + feature extraction + data transformation and analysis	Did not use machine learning for feature extraction, which limits the scope of the feature map
Fully automate the tongue contour extraction [6]	Traditional	MSD	1.01–0.63 mm	Tongue US images	Snake algorithm + phase symmetry filter + algorithm resetting	Computation complexity, too many constraints
Autotrace [104]	Machine Learning	MSD	0.73 mm	Tongue US images	Deep learning + translational deep belief network	High computation cost; limited training dataset
Enhanced Autotrace [105]	Machine learning	MSD	1.0 mm	Tongue US images	Deep autoencoder	Autoencoder has limited ability to classify features
CNN to automate the tongue segmentation [110]	Machine learning	MSD	U-net = 5.81 mm (Dense U-net) = 5.6 mm	Tongue US images	U-net + Dense U-net	U-net has limited generalization capability (Dense U-net) perform better than U-net but it is slower
BowNet and wBowNet [112]	Machine learning	MSD	1.4mm	Tongue US images	Deep network in landmarks	Lack of global feature extraction of the CNN
TongueNet [113]	Machine learning	MSD	0.31 pixel	Tongue US images	Multiscale contextual information + dilated convolution	Random selection of the annotated landmarks is not efficient in the method
DCAE-based B-Mode US [115]	Machine learning	Word error rate	6.17 %	Tongue US images	Denoising convolutional autoencoder (DCAE)	Autoencoder has a limitation of classifying features in latent space and difficult to be generalized in global context
ConvLSTM [116]	Machine learning	MSE and CW-SSIM	MSE = 17.13 CW-SSIM = 0.932	Tongue US images	CNN + LSTM	Limited memory, predicting up to nine future frames
U-NET and shape-consistency-based regularizer [117]	Traditional and machine learning	MSD	(2.243 ± 0.026) mm	Tongue US images	U-net architecture + temporal continuity using shape-consistency-based regularizer	Temporal continuity can be computational expensive for real-time applications
wUnet [118]	Machine learning	MSD	1.18 mm	Tongue US images	U-net architecture + VGG19 block instead of skip connections	VGG19 may add unnecessary features to the network and cause overfitting
SottoVoce [119]	Machine learning	Speech recognition success ratio	65%	Tongue US images + speech audio recording	Deep CNN	Acoustic sensor is not practical for smart systems integration

## Data Availability

Not applicable.

## Abbreviations

The following abbreviations are used in this manuscript:

2D	Two dimensions
3D	Three dimensions
ASM	Active shape model
CNN	Convolutional neural networks
CW	Complex wavelet
SSIM	Structural similarity index measure
CT	Computed tomography
DCAE	Denoising convolutional autoencoder
EV	Evaluation
EPG	Electropalatography
EMA	Electromagnetic articulatory
ECG	Electrocardiography
EEG	Electroencephalography
EMG	Electromyography
LSTM	Long short-term memory
MSD	Mean sum of distances
MSE	Mean squared error
MRI	Magnetic resonance imaging
ML	Machine learning
PMA	Permanent magnet articulator
PCA	Principal component analysis
RMSE	Root-mean-square error
SSI	Silent-speech interface
US	Ultrasound
